# Overexpression of GSE1 Related to Trastuzumab Resistance in Gastric Cancer Cells

**DOI:** 10.1155/2021/8834923

**Published:** 2021-02-02

**Authors:** Wenbin Wang, Shuanhu Wang, A. man Xu, Xiao Yuan, Liguo Huang, Jun Li

**Affiliations:** ^1^Department of Basic and Clinical Pharmacology, School of Pharmacy, Anhui Medical University, Hefei, Anhui, China; ^2^Hainan Weikang Pharmaceutical, Co., Ltd. Qianshan, Anhui, China; ^3^Inflammation and Immune Mediated Diseases Laboratory of Anhui Province, Hefei, Anhui, China; ^4^Department of General Surgery, The Fourth Affiliated Hospital of Anhui Medical University, Hefei, Anhui, China; ^5^Department of Gastrointestinal Surgery, The First Affiliated Hospital of Bengbu Medical College, Bengbu, Anhui, China

## Abstract

Gastric cancer is one of the most prevalent human cancers with poor prognosis. Trastuzumab is a well-used targeted drug for gastric cancer with HER2 amplification. Trastuzumab resistance restrains the clinical use of trastuzumab. In this study, we reported human Gse1 coiled-coil protein (GSE1) promoted trastuzumab resistance in HER2-positive gastric cancer cells. Acquired trastuzumab-resistant gastric cancer cells overexpressed GSE1, and depletion of GSE1 decreased the trastuzumab resistance of trastuzumab-resistant gastric cancer cells. BCL-2 was a downstream gene positively regulated by GSE1 and also performed promoting the role of trastuzumab resistance in HER2-positive gastric cancer cells. A high level of GSE1 was associated with a high risk of tumor lymph node metastasis and higher clinical stage in HER2-positive gastric cancer patients. GSE1 was a potential target that could be used for HER2-positive gastric cancer therapy.

## 1. Introduction

Gastric cancer is one of the most prevalent human cancers and has a high lethality worldwide [1, 2]. Surgical resection and chemotherapy are the main treatments for gastric cancer, but the survival rate of advanced gastric cancer patients is less than 1 year [3, 4]. HER2 (human epidermal growth factor receptor 2 (ERBB2)) amplification is found in 10-15% of gastric cancer patients, and trastuzumab is a monoclonal antibody drug that directly targets HER2 [5, 6]. HER2-targeted therapy could dramatically remit tumor progression. However, acquired drug resistance retarded the use of trastuzumab in gastric cancer [7]. As reported previously, NES1/KLK10 pathway [5], COL4A1 [7], microRNA-21/PTEN pathway [8], miR-223/FBXW7 pathway [9], etc. contributed to trastuzumab resistance of gastric cancer. The mechanisms involved in trastuzumab resistance of gastric cancer are complex; therefore, further study of the detailed molecular mechanisms of trastuzumab resistance in gastric cancer is desirable and urgent.

Human Gse1 coiled-coil protein (GSE1), also named KIAA0182, is a proline-rich protein. In Hakimi et al.'s study, they firstly isolated and identified GSE1 through an ion trap mass spectrometry [10]. GSE1 was reported to be an oncogene and was a direct target of miR-489-5p in human breast cancer cells [11]. In our previous study, we reported that GSE1 promoted proliferation and metastasis of human gastric cancer cells both *in vitro* and *in vivo*; a high level of GSE1 was associated with worse clinicopathological parameters including lymph node metastasis, histological grade, depth of invasion, and clinical stage in gastric cancer patients; overexpression of GSE1 was associated with decreased relapse-free survival rate and overall survival rate in gastric cancer patients [12]. For further study, we want to know the role of GSE1 in trastuzumab resistance of human gastric cancer.

In this study, we examined that forced expression of GSE1 promoted trastuzumab resistance in human HER2-positive gastric cancer cells. Acquired trastuzumab-resistant gastric cancer cells MKN45-HR and NCI-N8-HR cells were developed, and GSE1 was examined to be overexpressed in these two trastuzumab-resistant cells. Depletion of GSE1 eliminated the resistance of MKN45-HR and NCI-N8-HR cells to trastuzumab. BCL-2 was proved to be positively regulated by GSE1, and BCL-2 also played a promoting role in trastuzumab resistance of gastric cancer cells. GSE1 was associated with a high risk of tumor lymph node metastasis and higher clinical stage in HER2-positive gastric cancer patients. Therefore, GSE1 was an important biomarker in HER2-positive gastric cancer and could be used as a novel target for adjuvant therapy.

## 2. Materials and Methods

### 2.1. Cell Lines and Cell Culture

Human HER2-positive gastric cancer cell lines MKN-45 and NCI-N87 were used in this study. These two cells were obtained from ATCC (the American Type Culture Collection). MKN45 and NCI-N87 cells were continuously treated with 10 *μ*g/ml trastuzumab for at least 6 months until these two cell lines got trastuzumab resistance as determined by MTT assay. These two trastuzumab-resistant cell lines were designated as MKN45-HR and NCI-N87-HR, respectively. These cells were cultured in a 37°C, 5% CO2, and humidified atmosphere as recommended.

### 2.2. Plasmids and RNA Oligonucleotide Transfection

GSE1 overexpressing plasmid pIRESneo3-GSE1 was constructed as described in our former study [12]. Vector pIRESneo3 plasmid was used as control. shGSE1, 5′-GCCUACAUCUAUGAUGAGUTT-3′ was used for GSE1 depletion. Plasmids and shRNA transfection were performed by using lip2000 (QIAGEN) as described in our former study [12].

### 2.3. Western Blotting

Western blotting analysis was essentially carried out as described in our previous study [12]. Rabbit polyclonal antibody against GSE1 (1 : 1000, Proteintech Group, Inc., Chicago, USA) and mouse monoclonal antibody against *β*-actin (1 : 5000, Sigma) were used.

### 2.4. MTT Assay

We performed 3-(4,5-dimethylthiazol-2-yl)-2, 5-diphenyltetrazolium bromide (MTT) assay to evaluate cell viabilities. One thousand cells (or transfected cells) per well were seeded into 96-well plates, different doses of trastuzumab were added into the medium, and MTT examination (cell viability tested at OD570 nm) was performed after 6 days.

### 2.5. Three-Dimensional (3-D) Matrigel Cell Culture Assay

We performed a 3-D matrigel cell culture assay to evaluate 3-D cell growth condition essentially as described in former studies [13]. Briefly, 3000 cells per well were seeded into 24-well plates with matrigel. After 4 days, trastuzumab was added into the medium, and cell growth condition was examined after 6 days. For quantification analysis, Cell Counting Kit-8 (Dojindo, Japan) was used.

### 2.6. RT-Quantitative PCR (RT-qPCR)

RT-qPCR was carried out to examine GSE1, SOX2, CD44, BCL-2, C-myc, and P53 mRNA levels in this study. SYBR Green Master Mix (Applied Biosystems) was used for RT-qPCR testing as described in our former study [12]. GAPDH was tested as control. RT-qPCR primers used were GSE1, forward 5′-AGAGCACCA CCAGGCCAGGAC-3′ and reverse 5′-CGTGGGTGCAGCATGGAGC-3′; SOX2, forward, 5′-GGGAAATGGGAGGGGTGCAAAAGAGG-3′ and reverse, 5′-TTGCGT GAGTGTGGATGGGGATTGGTG-3′; CD44, forward, 5′-TCAGTCACAGACCTGC CCAA-3′ and reverse, 5′-CCTTTCTGGACATAGCGGGT-3′; BCL-2, forward, 5′-CGCCCTGTGGATGACTGAGT-3′ and reverse, 5′-GGGCCAAACTG AGCAGAGTC-3′; C-myc, forward, 5′-CAGGACTGTATGTGGAGCGGCTTC-3′ and reverse,5′-TGCTGTCGTTGAGAGGGTAG-3′; P53, forward, 5′-TGCAGC TGTGGGTTGATTCC-3′ and reverse,5′-AAACACGCACCTCAAAGCTGTTC-3′; and GAPDH, forward 5′-TGCACCACCAACTGCTTAGC-3′ and reverse 5′-GGCATGGACTGTGGTCATGAG-3′.

### 2.7. Cell Sphere Formation Assay

Cell sphere formation assay was carried out as described in a previous study [14]. Pictures were shot, and cell sphere numbers were counted.

### 2.8. Clinical Samples

Forty paraffin-embedded HER2-positive gastric cancer tissues were collected in the First Affiliated Hospital of Anhui Medical University (Hefei, Anhui, China) during 2017-1019. Patient pathological features including age, tumor size, tumor lymph node metastasis, histological grade, and clinical stage were collected. Local approval was got from the Institutional Review Boards of Anhui Medical University before work. We performed this study according to The Code of Ethics of the World Medical Association (Declaration of Helsinki). Informed consent was got from every patient.

### 2.9. Immunohistochemistry (IHC)

Protein levels of GSE1 in patient paraffin-embedded tissues were examined by immunohistochemistry (IHC), which was carried out as described in our former study [12]. Rabbit polyclonal antibody against GSE1 (1 : 100, Proteintech Group, Inc., Chicago, USA) was used. Positive-stained cells 10% or more than 10% were designated to be GSE1 high, and positive-stained cells less than 10% were designated to be GSE1 low.

### 2.10. Statistical Analyses

Each experiment was replicated at least 3 times, and the results showed in the figures represented the average. Unpaired two-tailed *t* test was used for statistical analysis in MTT assay, 3-D matrigel cell culture assay, RT-qPCR, and cell sphere formation assay. Pearson's chi-square test was used for statistical analysis in immunohistochemistry and patient pathological feature study. The differences were statistically significant when *P* < 0.05.

## 3. Results

### 3.1. Overexpressing of GSE1 Promoted Trastuzumab Resistance of Human HER2-Positive Gastric Cancer Cells

**Figure 1:**
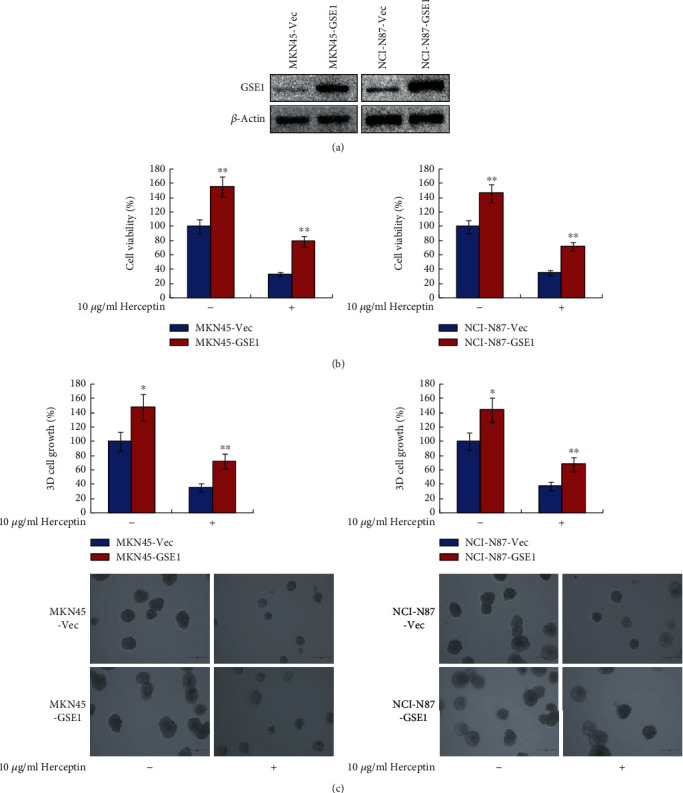
Overexpressing of GSE1 promoted trastuzumab resistance of human HER2-positive gastric cancer cells. MKN45 and NCI-N87 cells were transfected with GSE1 plasmids or control vector (designated as MKN45-GSE1, MKN45-Vec, NCI-N87-GSE1, and NCI-N87-Vec, respectively). (a) Protein levels of GSE1 were examined by western blotting. *β*-Actin was a negative control. (b) Cells were treated with 10 *μ*g/ml trastuzumab for 6 days, and cell viabilities were detected by MTT assay. (c) 3-D matrigel cell culture assay was performed to evaluated cell 3-D growth with trastuzumab treatment for 6 days. ^∗^*P* < 0.05. ^∗∗^*P* < 0.01.

In our former study, we have demonstrated that GSE1 promoted tumor growth and metastasis in gastric cancer cells and was associated with poor survival outcome in gastric cancer patients [12]. For further study, we examined the role of GSE1 in the trastuzumab response of human HER2-positive gastric cancer cells. GSE1 overexpressing plasmids or control Vec plasmids were transfected into HER2-positive gastric cancer cells MKN45 and NCI-N87, and these cells were indicated as MKN45-GSE1, MKN45-Vec, NCI-N87-GSE1, and NCI-N87-Vec, respectively. As shown in Figure 1(a), the protein levels of GSE1 were dramatically higher in MKN45-GSE1 and NCI-N87-GSE1 cells compared with MKN45-Vec and NCI-N87-Vec cells, respectively. As determined by MTT assay, cell viabilities increased significantly in both MKN45-GSE1 and NCI-N87-GSE1 cells compared with MKN45-Vec and NCI-N87-Vec cells. On exposure to trastuzumab, cell viabilities of MKN45-GSE1, MKN45-Vec, NCI-N87-GSE1, and NCI-N87-Vec all decreased; however, the cell viability decreases of MKN45-GSE1 and NCI-N87-GSE1 cells were much less compared with MKN45-Vec and NCI-N87-Vec cells, respectively (Figure 1(b)). Concordantly, as determined by 3-D matrigel cell culture assay, overexpression of GSE1 dramatically promoted 3-D cell growth of both MKN45 and NCI-N87 cells on exposure to10 *μ*g/ml trastuzumab (Figure 1(c)). Therefore, overexpressing of GSE1 promoted trastuzumab resistance in HER2-positive gastric cancer cells MKN45 and NCI-N87.

### 3.2. Trastuzumab-Resistant Gastric Cancer Cells Expressed Higher GSE1

**Figure 2:**
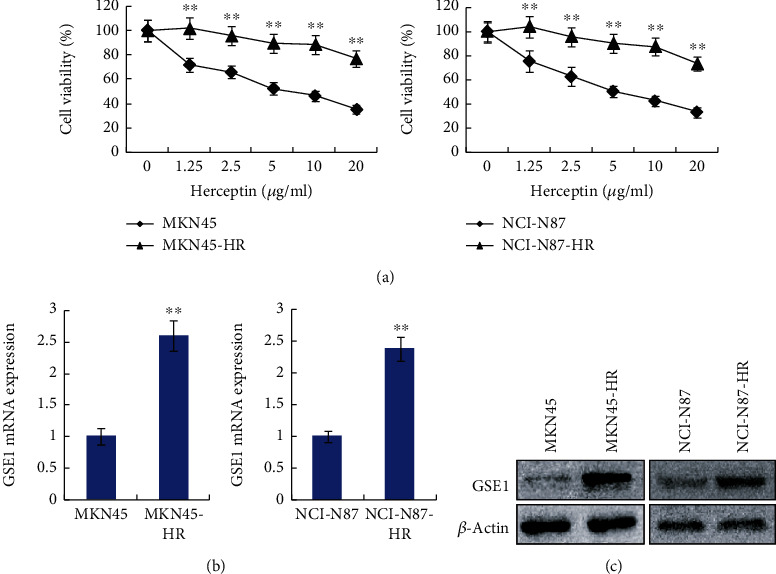
Trastuzumab-resistant gastric cancer cells expressed higher GSE1. (a) Cell growth curves of MKN45-HR, MKN45, NCI-N87-HR, and NCI-N87 cells under different doses of trastuzumab were examined by MTT assay. (b) mRNA levels of GSE1 in MKN45-HR, MKN45, NCI-N87-HR, and NCI-N87 cells were examined by RT-qPCR. GAPDH was used as a control. (c) Protein levels of GSE1 in MKN45-HR, MKN45, NCI-N87-HR, and NCI-N87 cells were examined by western blotting. *β*-Actin was a negative control. ^∗∗^*P* < 0.01.

Next, HER2-positive gastric cancer cells MKN45 and NCI-N87 were developed to trastuzumab-resistant cells, and they were indicated as MKN45-HR and NCI-N87-HR cells, respectively. As shown in Figure 2(a), both MKN45-HR and NCI-N87-HR cells showed significant trastuzumab resistance on exposure to10 *μ*g/ml or even 20 *μ*g/ml trastuzumab as determined by MTT assay. As examined by RT-qPCR, the mRNA levels of GSE1 were dramatically higher in both MKN45-HR and NCI-N87-HR cells compared with control parental MKN45 and NCI-N87 cells, respectively (Figure 2(b)). Moreover, as determined by western blotting assay, the protein levels of GSE1 were also much higher in MKN45-HR and NCI-N87-HR cells compared with MKN45 and NCI-N87 cells, respectively (Figure 2(b)). Therefore, trastuzumab-resistant gastric cancer cells expressed higher GSE1 compared with trastuzumab-sensitive cells.

### 3.3. Depletion of GSE1 Decreased the Trastuzumab Resistance in Trastuzumab-Resistant Gastric Cancer Cells

**Figure 3:**
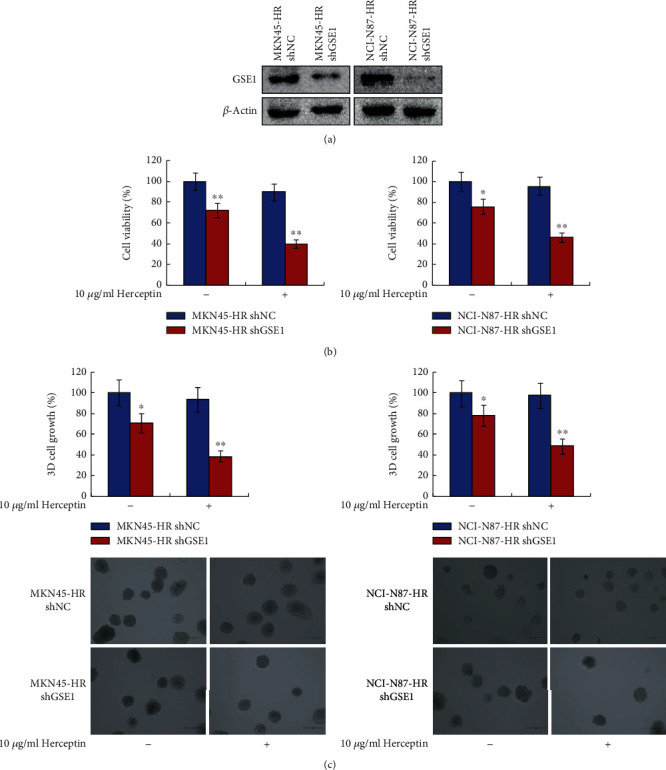
Depletion of GSE1 decreased the trastuzumab resistance in trastuzumab-resistant gastric cancer cells. MKN45-HR and NCI-N87-HR cells were transfected with shGSE1 or shNC (designated as MKN45-HR shGSE1, MKN45-HR shNC, NCI-N87-HR shGSE1, and NCI-N87-HR shNC, respectively). (a) Protein levels of GSE1 were examined by western blotting. *β*-Actin was a negative control. (b) Cells were treated with 10 *μ*g/ml trastuzumab for 6 days, and cell viabilities were detected by MTT assay. (c) 3-D matrigel cell culture assay was performed to evaluated cell 3-D growth with trastuzumab treatment for 6 days. ^∗^*P* < 0.05. ^∗∗^*P* < 0.01.

To examine the role of GSE1 in trastuzumab-resistant gastric cancer cells, GSE1 was depleted by GSE1-shRNA (designated as shGSE1) in MKN45-HR and NCI-N87-HR cells. As shown in Figure 3(a), the protein levels of GSE1 decreased significantly in both MKN45-HR shGSE1 and NCI-N87-HR shGSE1 cells compared with MKN45-HR shNC (negative control shRNA) and NCI-N87-HR shNC cells, respectively. As determined by MTT assay and 3-D matrigel cell culture assay, depletion of GSE1 dramatically decreased cell viabilities and 3-D cell growth on exposure to 10 *μ*g/ml trastuzumab in both MKN45-HR and NCI-N87-HR cells compared with control (Figures 3(b) and 3(c)). Therefore, depletion of GSE1 decreased the trastuzumab resistance of trastuzumab-resistant gastric cancer cells MKN45-HR and NCI-N87-HR.

### 3.4. BCL-2 Was Regulated by GSE1 and Contributed to Trastuzumab Resistance of Gastric Cancer Cells

**Figure 4:**
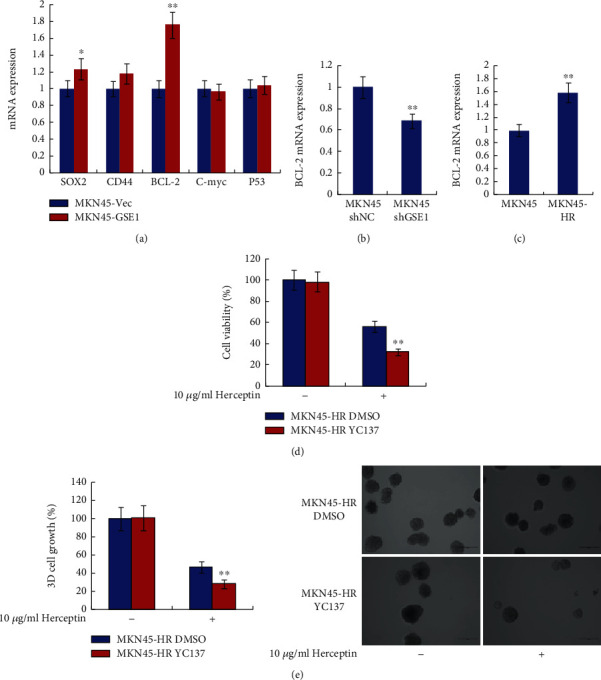
BCL-2 was regulated by GSE1 and contributed to trastuzumab resistance of gastric cancer cells. (a) mRNA levels of SOX2, CD44, BCL-2, C-myc, and P53 in MKN45-GSE1 and MKN45-Vec cells were examined by RT-qPCR. (b) mRNA levels of BCL-2 in MKN45 cells after transfection of shGSE1 or shNC were examined by RT-qPCR. (c) mRNA levels of BCL-2 in MKN45-HR and MKN45 cells were examined by RT-qPCR. For RT-qPCR, GAPDH was examined as control. (d) Cell viabilities of MKN45-HR cells ±10 *μ*g/ml trastuzumab and ±10 *μ*M YC137 were examined by MTT assay. (e) 3-D matrigel cell culture assay was performed in MKN45-HR cells ±10 *μ*g/ml trastuzumab and ±10 *μ*M YC137. ^∗^*P* < 0.05. ^∗∗^*P* < 0.01.

To unveil the downstream mechanisms involved in the trastuzumab-resistance promoting role of GSE1 in HER2-positive gastric cancer cells, several candidate genes were examined by RT-qPCR in MKN45-GSE1 and MKN45-Vec cells. These candidate genes included SOX2, CD44, BCL-2, C-myc, and P53. All of them were important oncogenes or tumor suppressors, participating in drug-resistance of gastric cancer, and they were reported to be associated with gastric cancer stem cell behaviors. As shown in Figure 4(a), the mRNA levels of BCL-2 were dramatically higher in MKN45-GSE1 cells compared with MKN45-Vec cells. However, there was no or less significant mRNA level change of the other 4 genes. To confirm this result, the mRNA levels of BCL-2 were examined in MKN45 shGSE1 and MKN45 shNC cells. As shown in Figure 4(b), the mRNA levels of BCL-2 were much lower in MKN45 shGSE1 cells compared with MKN45 shNC cells. Therefore, BCL-2 was positively regulated by GSE1 in gastric cancer cells. In addition, the mRNA levels of BCL-2 were also higher in MKN45-HR cells compared with parental MKN45 cells (Figure 4(c)). Next, BCL-2 inhibitor YC137 was introduced to examine the role of BCL-2 in trastuzumab resistance of gastric cancer cells. Functional inhibition of BCL-2 by YC137 dramatically decreased the trastuzumab resistance of MKN45-HR as determined by MTT assay and 3-D matrigel cell culture assay (Figures 4(d) and 4(e)). Therefore, BCL-2 was positively regulated by GSE1, and BCL-2 also promoted trastuzumab resistance of gastric cancer cells. The trastuzumab-resistance promoting role of GSE1 might be mediated by BCL-2 in HER2-positive gastric cancer cells.

### 3.5. GSE1 Promoted Cell Sphere Formation of MKN45 Cells

**Figure 5:**
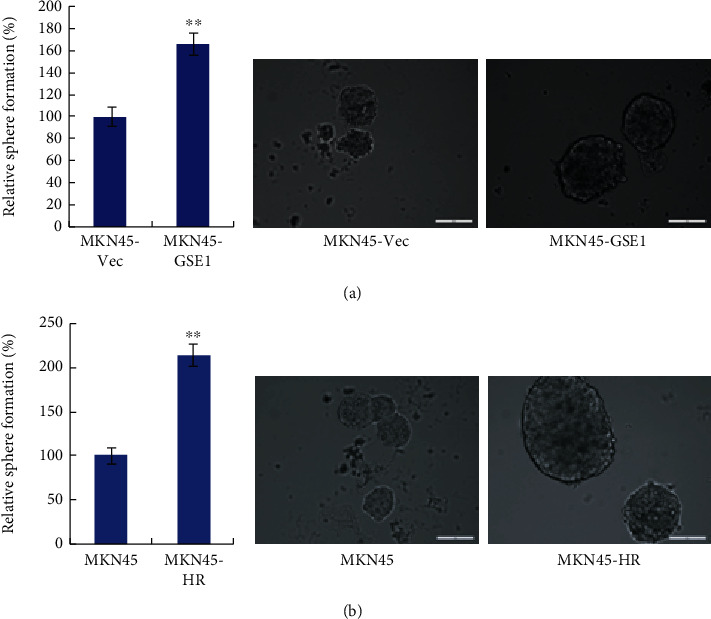
GSE1 promoted cell sphere formation of MKN45 cells. (a, b) Cell sphere formation assay was performed (a) in MKN45-GSE1 and MKN45-Vec cells and (b) in MKN45-HR and MKN45 cells. Representative pictures were shown. ^∗∗^*P* < 0.01.

For further study, we examined cell sphere formation in MKN45-GSE1 and MKN45-Vec cells. As shown in Figure 5(a), overexpression of GSE1 extremely promoted cell sphere formation of MKN45 cells. Moreover, the cell sphere number was significantly higher in MKN45-HR cells compared with parental control MKN45 cells (Figure 5(b)). Cell sphere formation was an important marker to show cancer stem cell behaviors. Therefore, the trastuzumab-resistance promoting role of GSE1 might be mediated by the promoting role of GSE1 in gastric cancer stem cell behaviors.

### 3.6. GSE1 Was Associated with Poor Pathological Features in HER2-Positive Gastric Cancer Patients

**Figure 6:**
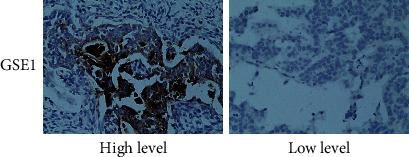
Expression levels of GSE1 in paraffin-embedded HER2-positive gastric cancer tissues. Protein levels of GSE1 in 40 paraffin-embedded HER2-positive gastric cancer tissues were examined by immunohistochemistry. Representative pictures were shown and the magnification was 400.

For further study, 40 paraffin-embedded HER2-positive gastric cancer tissues were collected, and the protein levels of GSE1 were examined by immunohistochemistry. According to the protein levels of GSE1 (Figure 6), these gastric cancer tissues were divided into the GSE1 high group and the GSE1 low group. The pathological features including patient age, tumor size, tumor lymph node metastasis, histological grade, and clinical stage of these 40 HER2-positive gastric cancer patients were collected, and association analysis was performed. As shown in Table 1, high levels of GSE1 were statistically correlated with a high risk of tumor lymph node metastasis (*P* = 0.014) and higher clinical stage (*P* = 0.037) in these 40 HER2-positive gastric cancer patients. However, there were no significant correlations between GSE1 levels and patient age, tumor size, or histological grade (all *P* > 0.05). Therefore, GSE1 was associated with poor pathological features in HER2-positive gastric cancer patients.

## 4. Discussion

In this study, we have observed that the forced expression of GSE1 in HER2-positive gastric cancer cells MKN45 and NCI-N8 dramatically enhanced the resistance to trastuzumab as determined by MTT assay and 3-D matrigel cell culture assay. In acquired trastuzumab-resistant cells MKN45-HR and NCI-N8-HR, we observed that both mRNA levels and protein levels of GSE1 were increased compared with parental cells MKN45 and NCI-N8. Depletion of GSE1 by shRNA dramatically decreased the resistance of MKN45-HR and NCI-N8-HR cells to trastuzumab as determined by MTT assay and 3-D matrigel cell culture assay. As reported previously, GSE1 promoted both cell proliferation and metastasis in breast cancer cells, and GSE1 was positively correlated with poor prognosis of patients with breast cancer [11]. In our former study, we have documented GSE1 was also oncogenic in human gastric cancer cells [12]. These results were concordant with our present results in this study, and herein, we further determined that GSE1 but also promote trastuzumab resistance in gastric cancer cells. Moreover, in this study, 40 HER2-positive gastric cancer tissues were collected, and the protein levels of GSE1 were examined. The results showed that high levels of GSE1 were associated with a high risk of tumor lymph node metastasis and higher clinical stage in these HER2-positive gastric cancer patients. As reported previously, GSE1 was observed to be overexpressed in gastric cancer tissues compared with normal gastric tissues; GSE1 positively associated with lymph node metastasis, histological grade, depth of invasion, and clinical stage in gastric cancer patients; GSE1 was positively correlated with worse survival rates in gastric cancer patients [12]. The former data also supported our present results. Therefore, GSE1 was an important biomarker in gastric cancer and especially in HER2-positive gastric cancer.

GSE1 was examined to positively regulate the expression of BCL-2 in HER2-positive gastric cancer cells. BCL-2 is also overexpressed in trastuzumab-resistant cells. Functional inhibition of BCL-2 by YC137 dramatically decreased the resistance of trastuzumab-resistant gastric cancer cells to trastuzumab. As reported previously, BCL-2 was a famous oncogene that suppressed apoptosis in human cancer cells [15, 16]. BCL-2 was oncogenic in gastric cancer [15, 17]. Moreover, BCL-2 was associated with methotrexate resistance in gastric cancer cells [18]. BCL-2 was a target of miR-1271 and miR-503 and mediated the suppressing role of miR-1271/miR-503 in cisplatin resistance of human gastric cancer cells [19, 20]. BCL-2 was also reported to promote multidrug resistance in gastric cancer cells [21, 22]. These reports were concordant with our present results. In addition, BCL-2 also mediated multidrug resistance in human breast cancer, lung cancer, colon cancer, etc. [23–25]. Moreover, BCL-2 was reported to be regulated by ARTN and contributed to trastuzumab resistance of human breast cancer [14]. Therefore, we herein proved that BCL-2 was also a promoter of trastuzumab resistance in HER2-positive gastric cancer cells. GSE1 positively regulated the expression of BCL-2, and the promoting role of GSE1 in trastuzumab resistance of HER2 positive gastric cancer might consequently be mediated by BCL-2.

In several publications, BCL-2 was determined to be an important promoter to regulate cancer stem cell-like behaviors in human breast cancer cells and consequently mediated multidrug (including trastuzumab) resistance of breast cancer cells [14, 26, 27]. Qi et al. reported that the overexpression of BCL2 and low expression of caspase 8 were related to TRAIL resistance of human brain cancer stem cells [28]. We doubted whether BCL-2 also mediated the promoting role of GSE1 in gastric cancer stem cell behaviors. Cell sphere formation assay was a method to evaluate cancer stem cell behaviors. We determined that forced expression of GSE1 increased cell sphere formation of gastric cancer cells. Moreover, cell sphere formation was significantly higher in trastuzumab-resistance gastric cancer cell MKN-HR compared with parental cell MNK45. Due to the limitation of experimental conditions, we did not perform a deeper study about the role of GSE1 in gastric cancer stem cell behaviors. If possible, we will perform this work in future. We made an immature conclusion that the promoting role of GSE1 in trastuzumab resistance of HER2-positive gastric cancer cells was partly through the promoting role of GSE1 in gastric cancer stem cell behaviors.

In summary, we have examined that GSE1 performed a promoting role in trastuzumab resistance of human HER2-positive gastric cancer cells. BCL-2 was a downstream gene that was regulated by GSE1. The promoting role of GSE1 in trastuzumab resistance of gastric cancer cells might be mediated by BCL-2. In HER2-positive gastric cancer patients, the overexpression of GSE1 was associated with poor pathological features. Therefore, GSE1 was a potential target that could be used for HER2-positive gastric cancer therapy.

## Figures and Tables

**Table 1:** Correlation between GSE1 levels and pathological features in HER2-positive gastric cancer patients.

		GSE1 levels (*n* (%))		*P*
Parameter	*n*	Low	High	
Age (years)				
≤60	15	6 (40.0)	9 (60.0)	
>60	25	10 (40.0)	15 (60.0)	1.000
Tumor size (cm)				
≤5	23	10 (43.5)	13 (56.5)	
>5	17	6 (35.3)	11 (64.7)	0.601
Lymph node metastasis				
No	18	11 (61.1)	7 (38.9)	
Yes	22	5 (22.7)	17 (77.3)	0.014
Grade				
I-II	24	9 (37.5)	15 (62.5)	
III	16	7 (43.8)	9 (56.2)	0.693
Stage				
I-II	17	10 (58.8)	7 (41.2)	
III-IV	23	6 (26.1)	17 (73.9)	0.037

## Data Availability

The data used to support the findings of this study are available from the corresponding author upon request.

## Abbreviations

ATCC: The American Type Culture Collection
GSE1: Gse1 coiled-coil protein
HER2: Human epidermal growth factor receptor 2
IHC: Immunohistochemistry
mRNA: Messenger RNA
MTT: 3-(4,5-Dimethylthiazol-2-yl)-2, 5-diphenyltetrazolium bromide
NC: Negative control
RT-qPCR: Reverse transcription-quantitative polymerase chain reaction
Vec: Vector
3-D: Three-dimensional.
